# Are Iranian Sulfur Mustard Gas-Exposed Survivors More Vulnerable to SARS-CoV-2? Some Similarity in Their Pathogenesis

**DOI:** 10.1017/dmp.2020.156

**Published:** 2020-05-18

**Authors:** Gholamreza Farnoosh, Mostafa Ghanei, Hossein Khorramdelazad, Gholamhossein Alishiri, Alireza Jalali Farahani, Alireza Shahriary, Seyed Reza Hosseini Zijoud

**Affiliations:** Applied Biotechnology Research Center, Baqiyatallah University of Medical Sciences, Tehran, Iran; Chemical Injuries Research Center, Systems Biology and Poisonings Institute, Baqiyatallah University of Medical Sciences, Tehran, Iran; Molecular Medicine Research Center, Research Institute of Basic Medical Sciences, Rafsanjan University of Medical Sciences, Rafsanjan, Iran; Department of Immunology, School of Medicine, Rafsanjan University of Medical Sciences, Rafsanjan, Iran; Trauma Research Center, Baqiyatallah University of Medical Sciences, Tehran, Iran; Clinical Research Development Unit, Imam Hossein Hospital, Shahid Beheshti University of Medical Sciences, Tehran, Iran

**Keywords:** COVID-19, Iran, mustard gas, SARS-CoV-2, Veteran

## Abstract

Coronavirus disease 2019 (COVID-19) is an infectious disease caused by the severe acute respiratory syndrome coronavirus 2 (SARS-CoV-2) that emerged as a health problem worldwide. It seems that COVID-19 is more lethal for Iranian veterans with a history of exposure to mustard gas. There are some similarities in the pathogenesis of SARS-CoV-2 and mustard gas in immune system disruption and pulmonary infection. SARS-CoV-2 and mustard gas inducing oxidative stress, immune system dysregulation, cytokine storm, and overexpression of angiotensin-converting enzyme II (ACE2) receptor in lungs that act as functional entry receptors for SARS-CoV-2. Moreover, Iranian survivors of mustard gas exposure are more susceptible and vulnerable to COVID-19. It is suggested that the principles of COVID-19 infection prevention and control be adhered to more stringently in Iranian survivors of mustard gas exposure than others who have not been exposed to mustard gas. Therefore, in this review, we discuss the different pathologic aspects of lung injury caused by mustard gas and also the relationship between this damage and the increased susceptibility of Iranian mustard gas exposed survivors to COVID-19.

## COVID-19 DISEASE AND ITS OUTBREAK

On March 11, 2020, the World Health Organization (WHO) announced a pandemic of the new outbreak of coronavirus disease 2019 (COVID-19) due to a severe acute respiratory syndrome coronavirus 2 (SARS-CoV-2), which began in December 2019 in Wuhan, China.^1^ As of March 25, 2020, a total of 198 countries and territories around the world and 1 international conveyance were affected, with 467,520 cases of COVID-19, and 21,174 deaths worldwide. Iran is also considered as the 25th country to be infected with SARS-CoV-2. As of March 25, 2020, there were 27,017 cases of infection (sixth, after China, Italy, United States, Spain, Germany), and 2077 deaths (fourth, after Italy, Spain, China) in Iran.^2^

Although scientists and researchers around the world are currently working to discover more scientific knowledge about SARS-CoV-2 and its pathogenesis, specific effective antiviral drugs against this novel coronavirus have not been reported and approved. Current management, therefore, includes travel restrictions, patient isolation, home quarantine, and supportive and symptomatic medical care.^3^

COVID-19 develops with the clinical presentation of pneumonia and infection, with an approximate incubation period of 2 to 14 days. In addition to respiratory symptoms, such as cough and shortness of breath, symptoms such as fever, myalgia, and fatigue have also been reported. The onset of the COVID-19 may rapidly progress to multiple organ damages, especially in the lung, presenting bilateral diffuse alveolar damage with cellular fibromyxoid exudates.^4^

The COVID-19 mortality rate is 4.3% worldwide, although in some countries, including Iran (7.8%) mortality is higher than worldwide.^2^ It has also been shown that people with underlying health issues, including respiratory disease, heart failure, hypertension, diabetes, immunodeficiency, transplant recipients, cancer, and old age are more vulnerable, which means that some people are at higher risk.^5^ Iranian survivors of mustard gas exposure seem to be another group that is at high risk of succumbing to SARS-CoV-2.

## PULMONARY COMPLICATIONS IN IRANIAN SURVIVORS OF MUSTARD GAS EXPOSURE

According to an epidemiological estimate, there are now more than 100,000 chemical warfare survivors in Iranian society who were exposed to mustard gas during the Iran-Iraq War (1980-88) and are now suffering from long-term complications, including ophthalmic, cutaneous, respiratory, and pulmonary problems.^6^ Respiratory and pulmonary problems are the leading cause of long-term disability in these survivors. In a study of 34,000 Iranian survivors of mustard gas exposure, 14,450 (42.5%) of them had pulmonary lesions. These pulmonary lesions were divided into 3 categories: mild (75%), moderate (15%), and severe (10%) based on pulmonary function tests (PFTs) and physical examination findings.^7^

Mustard gas has both acute (early) and chronic (late) effects on the respiratory system. Routine exposure to mustard gas leads to acute symptoms of the upper respiratory tract, such as irritated throat, sore throat, rhinorrhea, sneezing, and discomfort in the nose and sinuses. With more severe exposure to mustard gas, nasal mucosa bleeding, dry cough, and tracheobronchitis occurs. Acute lower respiratory symptoms usually include cough, shortness of breath, and burning sensation in the chest, hemoptysis, inflammation of the airways, and pneumonia and acute respiratory distress syndrome. Over time, these symptoms gradually become chronic.^6,8,9^

Based on chest radiograph (CXR) findings in survivors late-onset chronic pulmonary complications of mustard gas include: increased bronchovascular marking, air retention, bronchiectasis, pneumonic infiltration, and radiographic evidence of pulmonary hypertension.^10,11^ In 197 Iranian survivors, 10 years after severe mustard gas exposure, some pulmonary complications, such as chronic bronchitis (58%), asthma (10%), bronchiectasis (8%), airway obstruction by granulation tissue (9%), and pulmonary fibrosis (12%), had been reported.^12^

In many studies, in mustard-exposed survivors, chronic bronchitis has been reported as the most common late-onset respiratory event following exposure to mustard gas. Hypoxemia and hypercapnia are most commonly seen in moderate to severe cases, leading to core pulmonale and pulmonary hypertension in the severe stages of the complications. Airway infection with bronchopneumonia is also a common problem that often leads to septicemia.^10,12^

Increased airway sensitivity, characterized by typical attacks of dyspnea, wheezing, and nocturnal cough, and airflow obstruction patterns in spirometry have been reported between 4 weeks and 20 years after mustard gas inhalation. Bronchospasm attacks are typically triggered by lung infections, asthma, environmental allergens, and cold air.^12,13^

The direct effects of mustard gas on the mucosa of the bronchial wall and, more importantly, recurrent respiratory infections following the inhalation of mustard gas, are responsible for the development of bronchiectasis. As shown in a study of 40 Iranian survivors with severe late complications of mustard gas, the severity, and prevalence of bronchiectasis lesions tend to increase over time. These lesions usually begin bilaterally in the lower lobe and then progress to the middle lobe and lingula segment. In severe cases with extensive bronchiectasis, pulmonary hypertension, and eventually, core pulmonale may occur.^10,14^

Bronchoalveolar lavage (BAL) fluid analysis in patients who have inhaled mustard gas demonstrated that these patients have a persistent local inflammatory process in the lower respiratory tract resulting in pulmonary fibrosis, years after initial contact. Histopathologic evaluation of transbronchial lung biopsies in 73 mustard-exposed survivors showed variegated fibrosis, diffuse fibrosis, and an absence of fibrosis in 86%, 4%, and 10% of patients, respectively. Interstitial pneumonitis accounted for 97% of all fibrosis cases.^15,16^

## THE PATHOGENESIS OF MUSTARD GAS IN ACUTE AND CHRONIC COMPLICATIONS

Sulfur mustard (SM) or mustard gas, (C_4_ H_8_ C_l2_ S) (2,2′-dichloroethyl sulfide), is an alkylating agent. SM is absorbed through inhalation, the skin, the anterior surface of the eye, the lungs, and the gastrointestinal tract.^17^ As noted, due to the effect of SM on the body, its complications are divided into acute and chronic/delayed effects. Even 4 decades after the Iran-Iraq war in the 1980s, the deleterious effects of SM poisoning on survivors are still not fully understood. The damage with varying severity to multiple organs of the body has been reported for many years in Iranian SM exposed survivors.

After adsorption, SM undergoes intramolecular cyclization, leading to the formation of an ethylene episulfonium ion intermediate. The cyclic intermediate reacts by alkylating various types of electron-rich biological molecules and attacking and breaking DNA at specific nucleotides. As a result, it inhibits the synthesis of DNA, RNA, and protein. Although SM reacts with RNA, proteins, and phospholipids, the consensus idea is that it is a DNA alkylating agent and has an effective role in delaying healing. The most important alkylating site in mammals is the residual guanine nitrogen. Cell death due to DNA cross-linking is delayed until DNA replication and cell division. However, at higher cellular exposures, other mechanisms are important and accelerate cell death. Another mechanism that may be involved in acute SM injury is the reduction of nicotinamide adenine dinucleotide. Other potential mechanisms thought to be involved include the rapid inactivation of sulfhydryl-containing proteins and peptides, such as glutathione. Glutathione and other sulfhydryl compounds are important in maintaining the proper oxidation-reduction state of cellular components as well as in reducing reactive oxygen species in the cell. Therefore, glutathione and other sulfhydryl compounds can act to prevent peroxidation and loss of membrane integrity. Reducing glutathione produces reactive oxygen species. Acute damage to the mucous membrane, which occurs after exposure to SM, is probably due to 1 or more of these mechanisms producing necrosis and cell death, which is continued by erythema, pain, vesicles formation, blisters, ulcer, and impaired wound healing. In addition to the mentioned mechanisms, SM has other side effects on cells, such as mitosis inhibition (effects on the immune system and hematopoietic as well as epithelial and reproductive tissues), mutagenesis, carcinogenesis, and cholinomimetic effects.^18,19^

**FIGURE 1:**
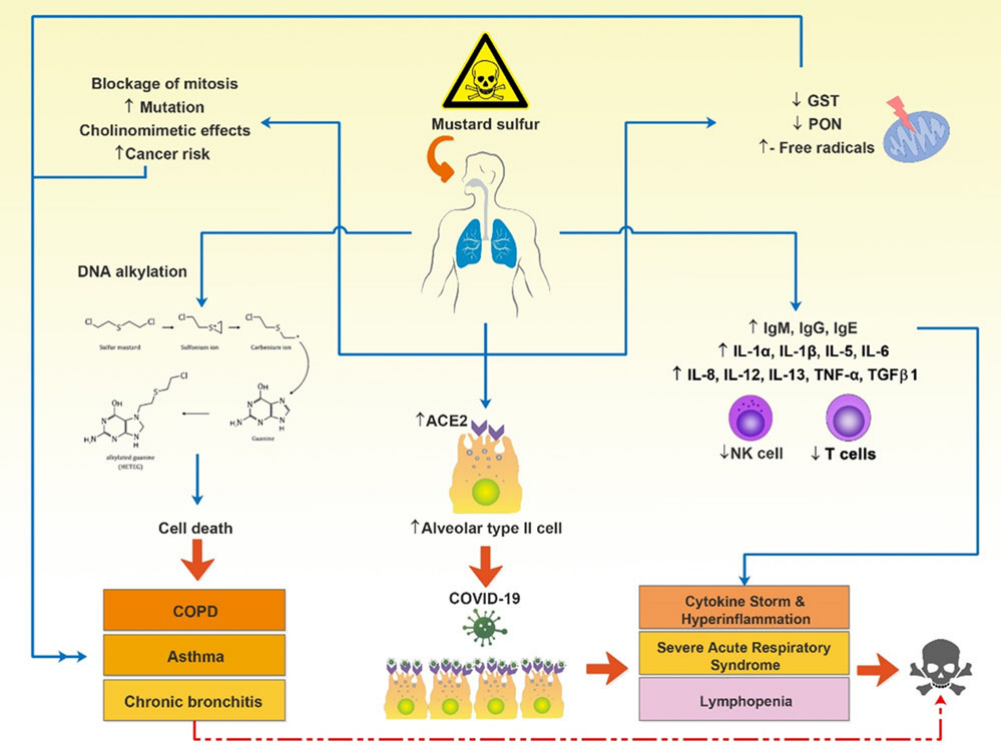
Different Adverse Effects of Mustard Gas on the Cellular and Molecular Mechanisms, Including the Activity of PON and GST Enzymes, the Number of Immune Cells, Production of Antibodies and Immune System Mediators, Mitosis, Alkylation of DNA, Expression of ACEII

The mechanism of delayed complications of SM poisoning is not well understood. A strong possibility is the production of reactive oxygen and nitrogen radicals in the cell that react with target molecules such as DNA, protein, lipid, and carbohydrate. The result of these reactions is the necrosis, inflammation, and alteration in the activity of antioxidant enzymes (superoxide dismutase, glutathione peroxidase, and catalase) producing more free radicals and exacerbating oxidative stress.^17,20^ A study in SM-exposed survivors reported a significant decrease in glutathione transferase and paraoxonase activity and mutation in the R genotype of paraoxonase.^21^ Decreased lung glutathione levels have been reported in survivors 20 years after SM exposure, which is directly related to altered pulmonary function. By identifying the role of free radicals in the acute effects of SM and activating pathways of its production in the body, this comes to mind that the late effects of exposure to SM, such as respiratory and pulmonary complications, can also be caused by the failure to control free radicals and continuous production of these compounds in the body. SM exerts its effects on the airways and lung parenchyma by free radicals and other agents. When it is absorbed by the respiratory system, SM causes inflammation of the tracheobronchial epithelium with severe leukocyte infiltration, alveolar hemorrhage with thrombus formation, and vacuolization of the lung parenchymal cells. The pathological changes that occur after exposure to SM are asthma, chronic obstructive pulmonary disease (COPD), and chronic bronchitis^22,23^ (Figure 1).

Additionally, systemic events and disorders caused by inhaled mustard gas are shown in this image. Abbreviations: ACEII, angiotensin-converting enzyme 2; COPD, chronic obstructive pulmonary disease; GST, glutathione S-transferases; NK cell, natural killer cell; PON, paraoxonase; TGF, tumor growth factor; TNF, tumor necrosis factor.

Evidence suggests that SM can also cause cellular and humoral immune dysfunction. Most SM-exposed survivors have been shown to have elevated levels of immunoglobulin (Ig) G and IgM during the first weeks to 6 months after exposure. Even at 8 years postexposure, the frequency of patients with elevated levels of IgG, IgE, and IgM remained significantly higher than the control group.^24^ Sixteen to 20 years after exposure, IgM levels were still significantly higher than controls.^10^

In addition, SM affects the immune system decreasing lymphocytes and neutrophils and increasing monocytes. These changes in the immune system in many people exposed to SM cause 2 major problems: a high incidence of malignancy and recurrent infections, among which acute myelocytic leukemia (AML) and acute lymphoblastic leukemia (ALL) are more common.^25,26^

BAL fluid analysis in patients with SM-exposed has shown an ongoing local inflammatory process that tends to develop pulmonary fibrosis many years after initial exposure. BAL fluid analysis also shows that pulmonary fibrosis after SM exposure is associated with increased inflammatory cytokines and chemokines, including interleukin (IL) -1α, IL-1β, IL-5, IL-6, IL-8, IL-12, IL-13, tumor necrosis factor-alpha (TNF-α), and tumor growth factor- beta1(TGF-β1).^27-29^

At the molecular level, SM releases cytokines, prostaglandins, matrix metalloproteinases, and serine proteinases^30^ (Figure 1).

Cellular immune suppression was reported in Iranian survivors 1, 2, and 3 years after SM exposure. Natural killer cells, an important component of cellular immunity, were significantly lower in patients suffering from severe respiratory complications 10 years as well as 16 to 20 years after exposure to SM.^31-33^

In another study, 7 specimens of lung biopsies were examined with an electron microscope at the WHO Research Center in Japan. Abnormal findings included proliferation, desquamation, and degeneration of the bronchial epithelial cells; interstitial fibrosis or fibrosing alveolitis; and an increased alveolar epithelial type I and type II cells as well as hyperplasia of ciliated and goblet cells.^34^

One possible mechanism of lung injury with SM is the pulmonary renin-angiotensin system, which plays a role in the development of inflammatory and fibrotic responses of the lung.^35^ Increased alveolar epithelial type I and type II cells have also been reported as abnormal findings in lung biopsy of SM-exposed.^34^ According to the findings of these studies, overexpression of alveolar epithelial type II cells (AECII) expressing a large amount of angiotensin-converting enzyme II (ACE2) in Iranian survivors of mustard gas exposure, make them a vulnerable and susceptible host for SARS-CoV-2 (Figure 1).

## PATHOGENESIS OF SARS-CoV-2

The prerequisite for entry of SARS-CoV-2 into the host cell is for the virus to bind to its specific receptor on the cell surface. This binding is mediated by the transmembrane spike glycoprotein (S) that forms homotrimers and protrudes from the viral surface. Spike can be broken down by proteases into an S1 subunit (containing the receptor-binding domain [RBD] and responsible for binding to the host cell receptor) and an S2 subunit (responsible for fusion of viral and cell membranes).^36^ Compared with other coronavirus proteins, the spike structural protein has the most variable amino acid sequence, which is the strongest option among all the coronavirus structural and nonstructural proteins to adapt to their hosts.^37^

Like SARS-CoV, the SARS-CoV-2 spike protein has a strong affinity for human ACE2, based on biochemical interaction studies and crystal structure analysis.^38^ It has also been directly shown that SARS-CoV-2 uses ACE2 as a cell entry receptor. It has been reported that residue 394 (glutamine) at RBD of SARS-CoV-2 can be recognized by lysine 31 on the human ACE2 receptor.^39^

After binding of the SARS-CoV-2 spike protein to ACE2, the spike protein is broken down via acid-dependent proteolysis by cathepsin, transmembrane protease serine 2 (TMPRSS2), or proteases furin. Consequently, the viral envelope merges with the cell membranes, allowing the virus to enter the host cell. ACE2 also plays a role in virus replication and spread in the host cell.^40,41^

In the normal human lung, ACE2 is mostly expressed on the surface of type I and II alveolar epithelial cells (AECI and AECII, respectively. In fact, 83% of ACE2-expressing cells are AECII, indicating that these cells can act as a reservoir for viral invasion.

Gene enrichment ontology analysis has shown that ACE2-expressing AECII have high levels of multiple viral process-related genes, including regulatory genes for viral processes, viral life cycle, viral assembly, and viral genome replication. These findings indicate that ACE2-expressing AECII facilitates the replication and spread of SARS-CoV-2 in the lung.^42^

Binding SARS-CoV-2 to ACE2 leads to overexpression of ACE2, that can damage alveolar cells, mostly due to increased inflammatory immune responses and cytokine storm leading to increased immune cell infiltration in the alveoli along with alveolar epithelial type II cells apoptosis. The damage to alveolar cells can, in turn, trigger a series of systemic reactions and even death. Notably, having a large area makes the lung highly susceptible to inhaled viruses, making it the most vulnerable target organ against SARS-CoV-2.^43,44^

The latest report indicates the infection of SARS-CoV-2 increases the expression levels of multiple proinflammatory cytokines, including interferon-gamma (IFN-γ), TNF-α, CCL2, and IL-6 in the serum, which suggests the inflammation storm may be involved in pulmonary inflammation in COVID-19. These results suggest that the excess immune cells migrating into lung tissue may cause an uncontrolled immune response, leading to the inflammation storm, and aggravated disease.^45^

Recent findings revealed that cluster of differentiation 147 (CD147) is a novel identified receptor for SARS-CoV-2 infection in binding with the spike protein. CD147 is a type I transmembrane glycoprotein expressed on epithelial cells. The glycoprotein is also highly expressed on activated T cells, which may facilitate the invasion of SARS-CoV-2 to lymphocytes by binding spike protein, suggesting that CD147 may be involved in lymphocytopenia. CD147 is also presented in activated inflammatory cells and participates in the regulation of cytokine secretion and leukocytes chemotaxis by binding with cyclophilin A (CyPA). CyPA is a proinflammatory cytokine that is up-regulated in response to virus infection.^45-51^

## LETHAL RISK OF SARS-COV-2 FOR SM-EXPOSED SURVIVORS

In SM-exposed survivors, in addition to producing free and active radicals and inducing oxidative stress, as well as impaired cellular and humoral immune function, and increased production of inflammatory cytokines and chemokines (cytokine storm) that damage the lungs, the AECI and AECII increase in number in the lung. It has been shown that lymphopenia, increased neutrophils, and increased levels of acute phase proteins such as C-reactive protein (CRP) are the most important laboratory findings in people with Covid-19.^40,46^ It has been previously mentioned that AECII express ACE2, and this makes them suitable hosts for SARS-CoV-2 that play an important role in virus entry and proliferation. Therefore, due to the increased number of AECII and the overexpression of ACE2 receptor induced by SM in SM-exposed survivors, these cells are more likely to be infected by SARS-Cov-2 and also more likely to have pulmonary complications and death (Figure 1).

## CONCLUSIONS

As described above, it is predicted that, in SM-exposed survivors, the respiratory and pulmonary clinical manifestations of COVID-19 will be more severe and have a shorter incubation period (from infection to clinical presentation), making SARS-CoV-2 more lethal in SM-exposed survivors. Therefore, it is suggested that more rigorous principles of infection prevention and control and special measures, such as isolation, should also be considered for SM-exposed survivors in hospitals. SM-exposed survivors must be more committed to the principles of health and adherence to home quarantine than others, because SM exposed survivors infected with COVID-19, have a much lower chance of surviving the associated respiratory and pulmonary symptoms.
